# Interactome analysis of myeloid-derived suppressor cells in murine models of colon and breast cancer

**DOI:** 10.18632/oncotarget.2489

**Published:** 2014-09-16

**Authors:** Alexander M. Aliper, Victoria P. Frieden-Korovkina, Anton Buzdin, Sergey A. Roumiantsev, Alex Zhavoronkov

**Affiliations:** ^1^ Federal Clinical Research Center of Pediatric Hematology, Oncology and Immunology, Moscow, Russia; ^2^ Insilico Medicine, Inc., Johns Hopkins University, Baltimore, MD, USA; ^3^ HiBiotechnology, LLC, Wellman, Iowa City, Iowa, USA; ^4^ Shemyakin-Ovchinnikov Institute of Bioorganic Chemistry, Miklukho-Maklaya, Moscow, Russia; ^5^ Pathway Pharmaceuticals, Limited, Wan Chai, Hong Kong; ^6^ Pirogov Russian National Research Medical University, Moscow, Russia; ^7^ Moscow Institute of Physics and Technology, Dolgoprudny, Moscow, Russian; ^8^ The Biogerontology Research Foundation, BGRF, London, UK

**Keywords:** MDSC, cancer, interactome, colon, suppressor, breast

## Abstract

In solid cancers, myeloid derived suppressor cells (MDSC) infiltrate (peri)tumoral tissues to induce immune tolerance and hence to establish a microenvironment permissive to tumor growth. Importantly, the mechanisms that facilitate such infiltration or a subsequent immune suppression are not fully understood. Hence, in this study, we aimed to delineate disparate molecular pathways which MDSC utilize in murine models of colon or breast cancer. Using pathways enrichment analysis, we completed interactome maps of multiple signaling pathways in CD11b+/Gr1(high/low) MDSC from spleens and tumor infiltrates of mice with c26GM colon cancer and tumor infiltrates of MDSC in 4T1 breast cancer. In both cancer models, infiltrating MDSC, but not CD11b+ splenic cells, have been found to be enriched in multiple signaling molecules suggestive of their enhanced proliferative and invasive phenotypes. The interactome data has been subsequently used to reconstruct a previously unexplored regulation of MDSC cell cycle by the c-myc transcription factor which was predicted by the analysis. Thus, this study represents a first interactome mapping of distinct multiple molecular pathways whereby MDSC sustain cancer progression.

## INTRODUCTION

Myeloid-derived suppressor cells (MDSC) are a heterogeneous group of progenitors of granulocytes, macrophages or dendritic cells (DC). Murine MDSC express selective cell surface markers CD11b and Gr1, which are used to distinguish them from other cell types [1]. Furthermore, differential expression of Gr1 is used to subdivide MDSC into a granulocytic group (CD11b+/Gr1 high) or a monocytic one (CD11b+/Gr1 low) and can be implemented for discovery of anti-neoplastic targets in murine models [1]. In physiological conditions, MDSC function to prevent immune-mediated damage to surrounding tissues in infection, chronic inflammation or in graft-versus-host disease [1, 2, 4]. In cancer however, MDSC infiltrate peritumoral tissues where they suppress CD4+ and CD8+ T-cells thus contributing to the immune tolerance [3]. Because of their functions, MDSC could even be implemented in differentiation systems for drug discovery in murine models [5].

Earlier studies suggest multiple mechanisms regulating MDSC proliferation and immune suppression. Granulocytic MDSC, for example, induce transitory suppression, which occurs via increases in arginase 1 (ARG1) and the reactive oxygen species (ROS) [6, 7]. To the contrary, monocytic MDSC irreversibly inhibit T-cells via activation of inducible nitric oxide synthase 2 with subsequent augmentation of reactive nitrogen species, in addition to activation of ARG1 [8-10]. Pro-inflammatory cytokines via STAT-3, -5 or -6 and c/EBPbeta transcription factors have been shown to promote ARG1 activation, ROS production and subsequent immune suppression [11-18].

GM-CSF, for example, is one cytokine that has been shown to modulate MDSC and immune responses in cancer in a dose-dependent manner. Namely, low GM-CSF levels enhance immune resistance whereas at higher levels MDCS proliferation and immune suppression occur [19-23]. Dose-dependent regulation of MDSC by the GM-CSF is attained via differential phosphorylation of JAK2 kinase with subsequent recruitment of distinct chaperone proteins [24-26]. In addition, components of PI3K and MAPK pathways may transduce GM-CSF signals [27, 28].

Hence, MDSC utilize multiple signaling cascades to expand their population. However, understanding the molecular networks where fine-tuning of these mechanisms occurs is lacking. In this study, we therefore aimed to compile a comprehensive picture of MDSC molecular networks in murine colon and breast cancers via generating MDSC interactome maps.

## RESULTS

In our research, we utilized GEO GSE21927 dataset originally derived from a study by Marigo et al where c26GM colon carcinoma or 4T1 breast carcinoma tumors were induced in BALB/c mice [29]. Our experimental groups, namely: 1) CD11b+ cells from spleens of c26GM colon cancer ; 2) CD11b+ cells from tumor infiltrates of c26GM colon cancer ; and 3) CD11b+ cells from tumor infiltrates of 4T1 breast cancer have been chosen based on the aforementioned dataset.

### Comparative analysis of differentially expressed MDSC genes

Total gene pool in each of the three experimental groups has been compared to normal controls in a search for differentially expressed genes. Genes with at least two-fold change in expression levels (p<0.05) were considered significantly regulated (Table 1). The threshold of 2 has been chosen deliberately based on: 1) the original study by Marigo et al. (2010) demonstrating prominent roles of C/EBPbeta gene in MDSC; and 2) our analysis showing increases in C/EBPbeta levels of 3.7-, 7.6- and 2.3-fold (p<0.05) in groups 1, 2 and 3 respectively [29].

We have found that CD11b+ splenic MDSC in c26GM colon cancer (group 1) possess 1041 differentially expressed genes with 364 up-regulated genes and 677 down-regulated genes (1, Table 1). The CD11b+ cells from c26GM tumor infiltrates (group 2) exhibit 1887 differentially expressed genes, 763 of which are up-regulated and 1124 are down-regulated (2, Table 1). Lastly, in 4T1 breast cancer the CD11b+ tumor-infiltrating MDSC (group 3) differentially express 2103 genes with 1081 genes being up-regulated and 1022 genes being down-regulated (3, Table 1).

**Table 1:** Comparative analysis of differentially expressed MDSC genes in three experimental groups.*,**

Group №	Total number of differentially expressed genes	Number of up-regulated genes	Number of down-regulated genes
1	1041	364	677
2	1887	763	1124
3	2103	1081	1022

* gene expression in each experimental group was compared to normal controls

** genes with a fold change ≥ 2 (p<0.05) were considered differentially expressed

### Complete interactome analysis of MDSC

A gene expression analysis thus suggests that MDSC possess unique tumor type-dependent profiles. However, given a complex post-transcriptional and post-translational gene regulation, these data are insufficient to extrapolate differential gene expression into distinct MDSC phenotypes.

We therefore subsequently utilized a highly annotated pathway analysis tool MetaCore™ to compile comprehensive interactome maps to elucidate these phenotypes (Supplemental Tables 1, 2 and 3). Specifically, the maps would allow for assessment of MDSC enrichment in molecular components of multiple signaling pathways, ligand-receptor complexes etc. Presented below are the selected segments of such analysis reflecting disparate functional contributions of several distinct classes of signaling molecules, namely transcription factors, kinases and proteases.

### Comparative analysis of MDSC enrichment in transcription factors

**Figure 1:**
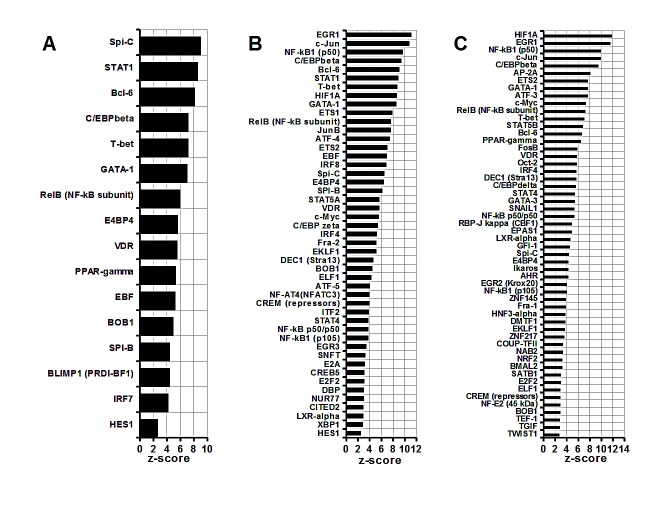
Comparative analysis of MDSC transcription factors (A) Splenic CD11b+ MDSC from c26GM colon cancer; (B) infiltrating CD11b+ MDSC from c26GM colon cancer; and (C) infiltrating CD11b+ MDSC from 4T1 breast cancer have been analyzed for an enrichment in transcription factors vs. healthy CD11b+ splenocytes using a pathway analysis tool MetaCore™. Higher z-scores (X axis) denote enhanced contributions (p<0.05, N=3 in each group).

Figure 1 illustrates a comparative analysis of enrichment in transcription factors in group 1 (Figure 1A), group 2 (Figure 1B) and group 3 (Figure 1C). It is noteworthy, that all three groups feature the C/EBPbeta (*C/EBPbeta*, Figure 1A, B and C) as one of the major regulators of MDSC function. This finding is in agreement with a study by Marigo et al. (2010) where the C/EBPbeta role has been confirmed experimentally [29]. In addition, interactome analysis suggests inputs from other transcription factors, which appear to be cell type-specific. In group 1, for example, SPI-C, STAT1, BCL-6 and C/EBPbeta are the most significant contributors to the MDSC homeostasis (*SPI-C, STAT1, BCL-6*, Figure 1A). Importantly, groups 2 and 3, when compared to normal controls, show greater numbers of differentially expressed transcription factors then a group 1 (Figure 1B). The most prominent roles in defining phenotype of MDSC in a group 2 are assigned to EGR1, c-jun and the components of NF-kappaB complex (*EGR1*, *c-jun, NF-KB1(p50),* Figure 1B). Other transcription factors, which may specifically define a phenotype of c26GM infiltrating CD11b+ MDSC (group 2), are HIF1A, STAT5A and c-myc (*HIF1A, STAT5A, c-myc*, Figure 1B). Similar to the group 1, BCL-6, STAT1 and C/EBPbeta-mediated pathways are suggested to provide significant contributions to their homeostasis (*C/EBPbeta*, *BCL-6, STAT1,* Figure 1B). In CD11b+ MDSC infiltrating 4T1 breast tumors (group 3, Figure 1C), HIF1A, EGR1, NF-kappaB1 and c-jun are the transcription factors with the highest z-scores (*HIF1A, EGR1, NF-kappaB1, c-jun,* Figure 1C). The c-myc dependent signaling plays more prominent role in these cells compared to a group 2 (*c-myc,* Figure 1B and C); several other transcription factors appear to be unique to the group 3, for example SNAIL1 or TWIST1 (*SNAIL1, TWIST1,* Figure 1C).

The interactome analysis of transcription factors therefore suggest their cell type- and disease type-specific contributions to a MDSC phenotype.

### Comparative analysis of MDSC enrichment in kinases

**Figure 2:**
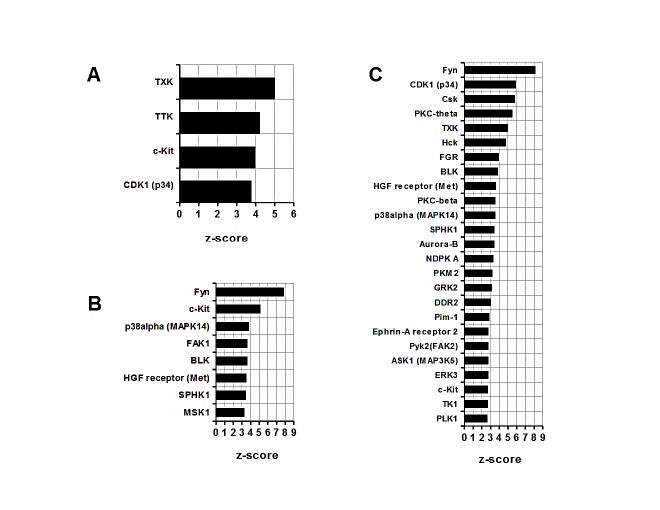
Comparative analysis of MDSC kinases (A) Splenic CD11b+ MDSC from c26GM colon cancer; (B) infiltrating CD11b+ MDSC from c26GM colon cancer; and (C) infiltrating CD11b+ MDSC from 4T1 breast cancer have been analyzed for an enrichment in kinases using a pathway analysis tool MetaCore™. Higher z-scores (X axis) denote enhanced contributions (p<0.05, N=3 in each group).

Similarly, functional impact of different classes of kinases has been assessed in groups 1, 2 and 3 (Figure 2A, B and C). A group 1 has been found to be significantly enriched in four kinases with TXK being assigned the highest z-score (*TXK*, Figure 2A). Unlike in a group 1, a group 2 features greater numbers of functionally important kinases with Fyn kinase predicted to have the most significant input (*Fyn*, Figure 2B). Similarly, in group 3, Fyn kinase occupies a first place on a z-score alignment list (*Fyn*, Figure 2C). In addition, many more other kinases appear to contribute selectively to the signal transduction in 4T1 infiltrating MDSC, for example PKC family or Aurora-B (*PKC-theta, PKC-beta, Aurora-B,* Figure 2C). Interactome analysis of kinases hence suggests an enrichment of distinct signaling pathways in different types of MDSC.

### Comparative analysis of MDSC enrichment in proteases

**Figure 3:**
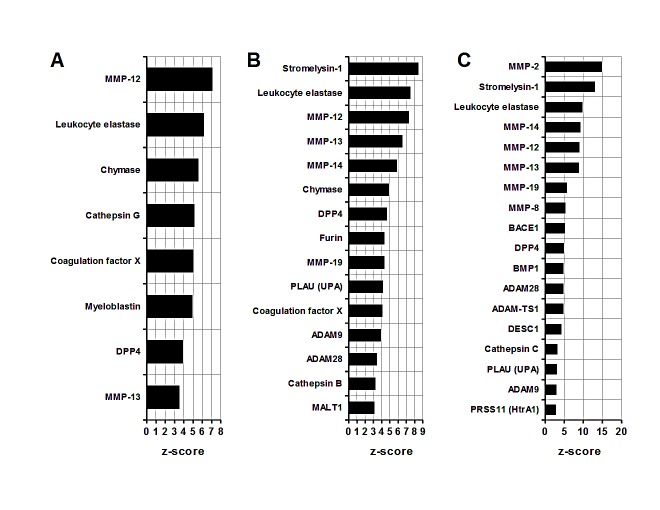
Comparative analysis of MDSC proteases (A) Splenic CD11b+ MDSC from c26GM colon cancer; (B) infiltrating CD11b+ MDSC from c26GM colon cancer; and (C) infiltrating CD11b+ MDSC from 4T1 breast cancer have been analyzed for an enrichment in proteases using a pathway analysis tool MetaCore™. Higher z-scores (X axis) denote enhanced contributions (p<0.05, N=3 in each group).

Proteases are molecules important in tissue remodeling and invasion. In the CD11b+ c26GM tumor splenocytes (group 1) MMP-12 (macrophage elastase) and a leukocyte elastase are predicted to have the greatest functional input among other proteases (*MMP-12, leukocyte elastase*, Figure 3A). The infiltrating MDSC from c26GM (group 2) and 4T1 (group 3) tumors show enrichment in matrix (stromelysin-1, MMPs) and intracellular (furin, ADAM family) metalloproteases (Figure 2B and C). A leukocyte elastase appears to be important for all three groups (*leukocyte elastase*, Figure 3A,) whereas stromelysin-1 (MMP-3) scores high on a scale of functional contributions selectively in infiltrating MDSC (*Stromelysin-1*, Figure 2B and). The analysis of protease-dependent pathways thus demonstrate enhanced contributions of different classes of metalloproteases in tumor-infiltrating MDSC compared to the cancer-associated splenocytes.

### Pharmacological inhibitors of functionally significant MDSC kinases and proteases

Results of the interactome analysis have been utilized to identify putative therapeutic compounds selectively targeting MDSC. Specifically, a virtual screening of selected MDSC proteases and kinases (Supplemental Tables 4 and 5 respectively) has been performed against a database of pharmacological inhibitors. Potential inhibitory effects on individual molecules have been prognosticated according to their z-scores and changes in gene expression. In addition, references to the earlier studies into the mechanisms of action of certain inhibitors have also been included into the results of screening.

### Reconstruction of c-myc-dependent signaling pathways in MDSC

**Figure 4:**
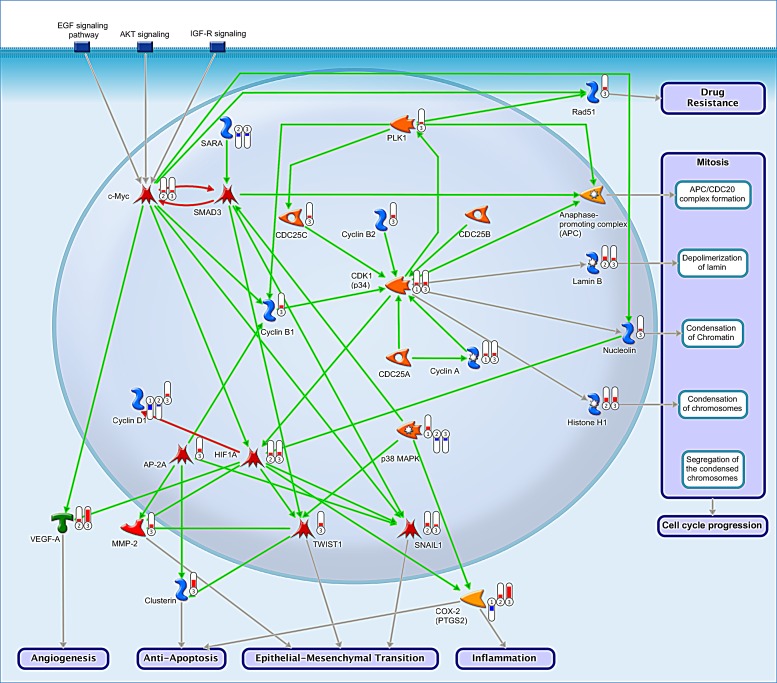
Reconstruction of putative c-myc-dependent signaling pathways in MDSC Circled numbers represent experimental groups 1, 2 and 3 respectively. The red bars above group numbers indicate an up-regulation whereas the blue bars represent down-regulation. The green, red and gray arrows denote activating, inhibitory and causative/unspecified interactions, respectively.

Our data indicate an importance of c-myc in infiltrating MDSC (groups 2 and 3). Given a limited knowledge regarding roles of this transcription factor in these cells, we reconstructed putative c-myc-dependent pathways in MDSC (Figure 4). The analysis suggests that deregulation of c-myc expression results from down-regulation of the SarA anchor proteins with subsequent inhibiton of SMAD signaling (Figure 4, *SARA, SMAD3, c-myc*). In contrast, in splenic CD11b+ cells (group 1) SMAD3 levels may be sustained due to the activity of p38 MAP kinase (Figure 4, *p38 MAPK*). Increased c-myc mediates several cellular processes, for example, progression through a cell cycle (Figure 4,*ell cycle progression*) or epithelial to mesenchimal transition (EMT) (Figure 4, *Epithelial-Mesenchimal Transition*). In mitosis, c-myc may stimulate formation of a cyclin B/CDK1 complex (Figure 4, *cyclin B1, cyclin B2, CDK1*) and a PLK1-dependent activation of APC complex (Figure 4, *PLK1, APC*). In addition, infiltrating MDSC but not the splenic ones appear to undergo EMT via a signaling cascade which involves a c-myc-dependent activation of HIF1A and subsequently TWIST and SNAIL (SNAI1) transcription factors (Figure 4, *HIF1A, TWIST, SNAIL*). Given increased levels of VEGF-A found by the interactome analysis in groups 2 and 3, it is predicted that this may occur due to an activation of c-myc (Figure 4, *VEGF-A, angiogenesis*).

## DISCUSSION

In this study, we apply an interactome analysis to explore unique molecular frameworks, which define CD11b+ MDSC from c26GM colon cancer and 4T1 breast cancer in mice. Compared to normal controls, infiltrating MDSC demonstrate enrichment in a larger number of multiple signaling molecules than do splenic c26GM cells, including transcription factors, kinases and proteases.

In-depth analysis of molecular pathways revealed by the interactome was not an objective of this study. However, an alignment by the z-score allows a comparative assessment of the relative functional contributions of these pathways to MDSC homeostasis in different types of cancer.

For example, a C/EBPbeta transcription factor appears to regulate both splenic and peritumoral MDSC in accordance with findings by Marigo et al (2010) [29]. To the contrary, other transcription factors, such as EGR1, c-jun, HIF1A or c-myc appear to selectively regulate MDSC in tumor infiltrates. Interestingly, we have earlier predicted a role for a c-jun proto-oncogene in MDSC epithelial to mesenchymal transition (EMT) [30]. This study indicates that infiltrating MDSC additionally utilize other EMT-related factors, namely TWIST and SNAIL (SNAI1) activated by the c-myc and HIF1A transcription factors (Figure 4). It is therefore possible to hypothesize that the EMT-induced invasion and migration in part define their phenotype [31]. Further analysis of pathway activation profiles using appropriate software such as OncoFinder would be required to advance this hypothesis [32].

Similarly, infiltrating MDSC in both breast and colon cancers have been predicted to be highly enriched in the EGR-1-regulated signaling. Importantly, earlier research has not defined a role for EGR1 in MDSC although it has been shown to induce transcription of a matrix metalloprotease 9 gene in tumor microenvironment [33]. Given the augmented contributions from multiple metalloproteases in our analysis, it is possible to suggest a novel EGR1-metalloprotease molecular network to regulate MDSC invasion [34].

Interestingly, a HIF1A transcription factor selectively regulates infiltrating MDSC but does not contribute to the homeostasis of CD11b+ cells from spleens in c26GM colon cancer. Contrary to our findings, using a different cancer model Corzo CA et al. (2010) have shown that hypoxia up-regulates HIF1A levels in splenic MDSC to enhance their immunosuppressive properties [35]. In addition, hypoxia and HIF1A induce differentiation of infiltrating MDSC into tumor-associated macrophages [35]. Normal peripheral mononuclear cells acquire MDSC phenotype following co-incubation with tumor cells via increases in HIF1A expression [36]. It is possible that selective enrichment of infiltrating MDSC in HIF1A found in our study suggests tumor type-specific mechanisms of immune suppression.

Both c26GM and 4T1 infiltrating MDSC have been found to be enriched in higher numbers of several classes of kinases compared to normal controls than the c26GM splenocytes. Data indicate that a proto-oncogene Fyn kinase provides most significant regulatory inputs in these cells. Similar to the aforementioned EGR-1 transcription factor, a role for Fyn kinase in MDSC is not well defined. It has been suggested to promote proliferation and anti-apoptosis in different types of cancer and thus may mediate MDSC expansion [37-39].

In conclusion, an interactome analysis is a powerful tool in delineating comprehensive molecular networks that define MDSC in different types of cancers.

## MATERIALS AND METHODS

*Experimental groups* were originally defined in GEO GSE21927 dataset by Marigo et al [29]. Briefly, c26GM colon carcinoma or 4T1 breast carcinoma tumors were induced in BALB/c mice [29]. Subsequently, the CD11b+ cells populating spleens and tumor infiltrates of diseased animals were analyzed using Affymetrix GeneChip MOE 430 arrays [29]. For present study, we have selected three experimental groups out of GEO GSE21927 dataset, namely: 1) CD11b+ cells from spleens of c26GM colon cancer (N=3); 2) CD11b+ cells from tumor infiltrates of c26GM colon cancer (N=3); and 3) CD11b+ cells from tumor infiltrates of 4T1 breast cancer (N=3). A group comprising the CD11b+ splenocytes from healthy BALB/c mice was used as a control (N=3) [29].

### Statistical analysis

Raw microarray data from GEO GSE21927 were normalized using a cytosine guanine robust multi-array analysis (GCRMA) algorithm and summarized using redefined probe set definition files from Brainarray repository (Version 17) [40]. A case-control pairwise comparison has been performed by comparing gene expression profiles of each experimental group to those of a control group. Empirical Bayes moderated t-test was performed using a Linear Models for Microarray Data (“limma”) package available for R statistical analysis (version 2.15.3; http://www.r-project.org/) [41]. Subsequently, a list of statistically significant differentially expressed genes has been obtained following the FDR adjustment of the resulting p-values at level of 0.05 and calculating mean fold-changes (FC) [42]. The genes with FC≥ 2 were denoted as significantly differentially expressed.

### Pathway enrichment analysis and interactome maps

A highly annotated automatic pathway analysis tool MetaCore™ (Thompson Reuters, New York, USA) has been utilized to perform pathways enrichment and the interactome analysis. Functional impact of an individual gene was estimated as a function of the number of interactions with other elements in the signaling network. Specifically, each gene has been predicted to regulate a certain number of downstream molecules based on mean values derived from hypergeometric distribution (*Expected* value). Genes found to have greater numbers of differentially expressed target molecules (*Actual* value) than predicted means (*Expected* value) were defined as “over-connected”, i.e. having larger than expected functional input. *Z-scores* were subsequently used to assess the enrichment in components of a particular pathway, with higher scores denoting pathways with greater magnitude of functional contributions.

Results of interactome analysis of experimental groups 1, 2 and 3 have been compiled into the corresponding tables (Supplemental Tables 1, 2 and 3). Parameters presented in the tables are as follows: *FC:* fold-change; *Actual: a* number of significantly differentially expressed genes regulated by the molecule of interest; *n:* a total number of significantly differentially expressed genes recognized by MetaCore™; *R:* a number of targets in the complete MetaCore™ database; *N:* a total number of gene-based objects in the complete MetaCore™ database; *Expected:* a mean value calculated from hypergeometric distribution (n*R/N), *Ratio:* a connectivity ratio (Actual/Expected); *z-score:*(Actual-Expected)/sqrt(variance); *p-value:* a probability to have the given value of Actual or higher (or lower for negative z-score).

Selected subsets of these analysis, namely significantly enriched (p<0.05) transcription factors, kinases and proteases were aligned by their respective z-scores and plotted. Virtual screening of proteases and kinases against a database of pharmacological inhibitors have been performed using a MetaCore™ Drug Lookup tool.

## SUPPLEMENTARY MATERIAL AND TABLES












