# Supplementary material for: Interactome analysis of myeloid-derived suppressor cells in murine models of colon and breast cancer
Source: Oncotarget. 2014 Sep 16;5(22):11345–53. doi: 10.18632/oncotarget.2489 (PMC4294358; doi:10.18632/oncotarget.2489)
Supplement: Supplementary file 1 [file oncotarget-05-11345-s001.pdf]

Interactome analysis of myeloid-derived suppressor cells in murine models of colon and breast cancer

Supplementary Material

Supplemental table 1: Complete interactome of CD11b+/Gr1 (high/low) MDSC from spleens of mice bearing c26GM colon carcinoma tumors (group 1).

Supplemental table 2: Complete interactome of CD11b+/Gr1 (high/low) MDSC from tumor infiltrates of mice bearing c26GM colon carcinoma tumors (group 2).

Supplemental table 3: Complete interactome of CD11b+/Gr1 (high/low) MDSC from tumor infiltrates of mice bearing 4T1 breast carcinoma tumors (group 3).

Supplemental table 4: Small molecule inhibitors of functionally important proteases in MDSC in groups 1, 2 and 3.

Supplemental table 5: Small molecule inhibitors of functionally important kinases in MDSC in groups 1, 2 and 3.
