# Supplementary material for: Interactome analysis of myeloid-derived suppressor cells in murine models of colon and breast cancer
Source: Oncotarget. 2014 Sep 16;5(22):11345–53. doi: 10.18632/oncotarget.2489 (PMC4294358; doi:10.18632/oncotarget.2489)
Supplement: Supplementary file 5 [file oncotarget-05-11345-s005.pdf]

Supplemental table 4: Pharmacological inhibitors of functionally important MDSC proteases.

Protein	§z-score/§§gene expression			Inhibitors	PMID Reference
	Group 1	Group 2	Group 3		
MMP-2	NS/ -1.09	NS/ 1.86	34.77/ 3.19	**S3304 extracellular region**	9484512 11749499 17404091
				**ONO4817 extracellular region**	10858013
				**Batimastat extracellular region**	9548812 9873712 11749499 12370049 12877590 15582436 16483784
				**Marimastat extracellular region**	10397503 10522712 11133099 11212095 12014967 12057646
				**Tanomastat extracellular region**	11060801 11139426 11749499 11923519
				**Rebimastat extracellular region**	11731431 11749499 12370049
				**Curcumin intracellular**	17197075
				**PG-116800 extracellular region**	16428338
				**ABT518 extracellular region**	11754593
				**Tetracycline extracellular region**	17267227
				**Clodronic acid intracellular**	17583333
				**TM601**	12454020
				**Oleic acid extracellular region**	11278959

				**Disulfiram intracellular**	14573756<br>18047805
				**Apratastat extracellular region**	
				**Doxycycline intracellular**	14681644<br>15090411<br>19885578<br>20126997<br>22487368
				**CTS-1027 extracellular region**	11133099<br>11549451<br>11749499,<br>16220987
				**Marimastat extracellular region**	10397503<br>10522712<br>11133099<br>11212095<br>12057646
				**ONO4817 extracellular region**	10858013
MMP-13	3.51/<br>-3.41	6.52/<br>5.68	8.83/<br>62.4	**Tanomastat extracellular region**	11060801<br>11749499
				**Batimastat extracellular region**	12877590
				**Doxycycline intracellular region**	19335556<br>19813262<br>15387968<br>17293464<br>17581617<br>10211886<br>10366106<br>12824233<br>15557436
				**PG-116800 extracellular region**	16428338
				**Apratastat extracellular region**	
				**Rebimastat extracellular region**	11749499<br>12370049
				**Tetracycline extracellular region**	9437511<br>17267227
				**CTS-1027 extracellular region**	11133099<br>11549451<br>11749499<br>16220987
				**Rivaroxaban extracellular region**	17116766

				**Fondaparinux extracellular region**	11715834
Coagulation factor X	5.01/ 10.87	4.09/ 9.83	NS/ 5.88	**Apixaban intracellular**	17379841
				**CS3030 extracellular region**	
				**Idraparinux extracellular region**	7757423<br>14717965
				**Otamixaban extracellular region**	11562341<br>11899252<br>12039587
				**Efegatran extracellular region**	11738569<br>12372529
				**Argatroban extracellular region**	16363236
				**PCI-27483 extracellular region**	
				**Melagatran extracellular region**	16363236
				**Amiloride extracellular region**	15115382
				**Heparin extracellular region**	9873490
				**Bivalirudin**	16363236
				**LY517717 extracellular region**	17408408
				**Dabigatran extracellular region**	11960487<br>15544461
				**Tanomastat extracellular region**	11060801<br>11139426<br>11749499
				**Tiludronic acid intracellular**	10960024
Stromelysin-1	NS/ 1.06	8.47/ 12.14	12.99/ 10.37	**Acetohydroxamic Acid extracellular region**	
				**Batimastat extracellular region**	9548812<br>9873712<br>11749499<br>12370049<br>12877590
				**Rebimastat extracellular region**	11731431<br>11749499<br>12370049
				**Tetracycline extracellular**	17267227

				**region**	
				**Marimastat extracellular region**	10397503<br>10522712<br>11212095<br>12057646
				**Alendronic acid extracellular region**	
				**Clodronic acid intracellular region**	17583333
				**CTS-1027 extracellular region**	11749499<br>16220987
				**PG-116800 extracellular region**	16428338
				**WXUK1 extracellular region**	15841327
				**Efegatran extracellular region**	10509932
PLAU (UPA)	NS/ 8.11	4.11/ 4.09	3.08/ 8.9	**Upamostat extracellular region**	15841327
				**Tolfenamic acid intracellular**	16820913
				**Amiloride extracellular region**	3106085<br>15115382
				**Aminocaproic Acid extracellular region**	
				**Tecogalan extracellular region**	11015037
				**Tranexamic acid extracellular region**	
				**Geldanamycin intracellular**	15978816
				**Incyclinide extracellular region**	11172680
				**Batimastat extracellular region**	11749499<br>12877590<br>15582436
MMP-14	NS/ -1.01	5.81/ 4.05	9.25/ 10.53	**Rebimastat extracellular region**	11731431
				**Marimastat extracellular region**	11749499<br>12057646
				**Apratastat extracellular region**	

				**Marimastat extracellular region**	12057646
				**ONO4817 extracellular region**	10858013
MMP-12	7.08/ 4.03	7.28/ 3.85	8.87/ 9.81	**Batimastat extracellular region**	
				**Acetohydroxamic Acid extracellular**	
				**AZD1236 extracellular region**	
				**Clodronic acid intracellular**	17583333
				**CTS21166 extracellular region**	20661410
				**Epigallocatechin-3-gallate extracel**	14592472 16392663
BACE1	NS/ -1.04	NS/ -1.36	5.15/ 3.08	**NO intracellular**	21371311 21371311
				**(2R,3R)-Epicatechin intracellular**	14592472
Furin	NS/ 1.59	4.33/ 3.34	NS/ 1.27	**Alpha-1 antitrypsin**	12006600
PRSS11 (HtrA1)	NS/ 1.42	NS/ 1.25	2.7/ 27.67	**Heparin extracellular region**	15534875

§ - $p<0.05$
§§- fold change compared to normal controls
NS – not significant ($p>0.05$)
