# Supplementary material for: Interactome analysis of myeloid-derived suppressor cells in murine models of colon and breast cancer
Source: Oncotarget. 2014 Sep 16;5(22):11345–53. doi: 10.18632/oncotarget.2489 (PMC4294358; doi:10.18632/oncotarget.2489)
Supplement: Supplementary file 6 [file oncotarget-05-11345-s006.pdf]

Supplemental table 5: Pharmacological inhibitors of functionally important MDSC kinases.

Protein	[§]z-score/[§§]gene expression			Inhibitors	PMID Reference
	Group 1	Group 2	Group 3		
CDK1 (p34)	3.77/ 2.4	NS/ 1.41	5.89/ 3.8	**Riviciclib**	17363486
				**AT7519M**	19174555<br>20101221
				**Alvocidib**	10843211<br>11032968<br>11063609<br>12190313<br>12593668<br>12643928<br>15125971<br>15689157<br>15780638<br>16250643<br>18639462
				**R547**	17064073
				**Seliciclib**	9046330<br>11032968
				**CP673451**	15705896
				**Quercetin**	11032968
				**O6-Benzylguanine**	12139449
				**BMS-387032**	15027863
				**Miltefosine**	8691459
				**CT327**	10434949
				**AEE788**	15256466
				**Roniciclib**	22821149
				**Linifanib**	16648571
				**Genistein**	11032968
				**CEP5214**	14640546
				**PD153035**	9357527
HGF receptor (Met)	NS/ 1.16	3.51/ 2.17	3.59/ 2.23	**Foretinib**	19808973<br>19808973
				**Tivantinib**	19318488<br>20484018<br>21454604
				**Crizotinib**	17483355

					21812414<br>17483355
				**Cabozantinib**	21926191<br>21926191
				**CP673451**	15705896
				**AEE788**	15256466
				**Pazopanib**	17620431<br>22037378
				**Pelitinib**	10973323
				**Vandetanib**	12477352
				**CT327**	12118367<br>12118367<br>14559966
TK1	NS/<br>1.47	NS/<br>-1.67	2.69/<br>2.22	**Zidovudine cytoplasm**	16828556<br>16831554
				**Trifluridine**	11425574
				**Brivudine**	16828556<br>7097717
				**Ibacitabine**	458818
				**Stavudine**	7097717
				**Thymidine cytoplasm**	4736965<br>7108896<br>10464024<br>12127539<br>12190323<br>15336255<br>16828556<br>16831554
				**Sorivudine**	11392548<br>17324575
				**Alovudine**	8381182<br>16828556<br>16831554
Aurora-B	NS/<br>1.56	NS/<br>-1.14	3.43/<br>2.26	**ENMD-2076 intracellular**	20560971<br>21177375
				**TAK-901 intracellular**	
				**AT9283**	19143567
				**Tozasertib**	14981513<br>15630414<br>16885368<br>18183025<br>22037378
				**CYC116**	20462263
				**SNS314**	19649632
				**Danusertib**	17125279
Pim-1	NS/	NS/	2.88/	**SGI-1776**	19734450

	2.28	2.56	2.33	**Masitinib**	22037378
				**Tivantinib**	20484018
				**Quercetin**	16302800
				**Mitoxantrone**	23442188
				**Genistein**	16302800
				**Alvocidib**	15711537
				**LY294002**	15657054
PLK1	NS/ 2.22	NS/ 1.45	2.6/ 2.29	**BI-2536**	17291758<br>18005335
				**Quercetin**	15853646
				**Rigosertib**	15766665<br>16223707<br>19474163<br>21812421<br>23103095
				**LY294002**	15664519
				**Volasertib**	19383823
DDR2	-1.07	1.58	3/5	**Masitinib**	22037378
				**Sorafenib**	18183025<br>22037378
				**Vandetanib**	22037377<br>22037378
Ephrin-A receptor 2	NS/ 1.39	NS/ 2.3	2.76/ 2.51	**Dasatinib**	18797457
				**Vandetanib**	15711537<br>22037377<br>22037378
				**Saracatinib**	19393585
PKM2	NS/ 1.42	NS/ 1.88	3.2/ 2.1	**CAP232**	17308100

§ - $p<0.05$

§§- fold change compared to normal controls

NS – not significant ($p>0.05$)
